# Dynamic multistimuli-responsive reversible chiral transformation in supramolecular helices

**DOI:** 10.1038/s41598-018-29152-9

**Published:** 2018-07-25

**Authors:** Santosh Goskulwad, Duong Duc La, Mohammad Al Kobaisi, Sidhanath V. Bhosale, Vipul Bansal, Ajayan Vinu, Katsuhiko Ariga, Sheshanath V. Bhosale

**Affiliations:** 1 Polymers and Functional Materials Division, Academy of Scientific and Innovative Research (AcSIR), CSIR-Indian Institute of Chemical Technology, Hyderabad, 500007 Telangana India; 2 School of Science, RMIT University, GPO Box 2476, Melbourne, VIC3001 Australia; 3 Ian Potter Nano BioSensing Facility and Nano Biotechnology Research Laboratory, RMIT University, GPO Box 2476, Melbourne, VIC 3001 Australia; 4 Global Innovative Centre for Advanced Nanomaterials, Faculty of Natural Built Environment and Engineering, University of Newcastle, Callaghan, Newcastle, 2308 NSW Australia; 5 WPI-MANA, National Institute for Materials Science (NIMS), 1-1 Namiki, Tsukuba, Ibaraki 305-0044 Japan; 6 Department of Advanced Materials Science, Graduate School of Frontier Sciences, The University of Tokyo, 5-1-5 Kahiwanoha, Kashiwa, Chiba 277-8561 Japan; 7 Department of Chemistry, Goa University, Taleigao Plateau, Goa, 403206 India

## Abstract

The design of new chiral chromophores that allow tunable assembly of higher order helical structures by using natural stimuli offers promising avenue in understanding various biological processes. In particular, access to dynamic multistimuli-responsive systems can provide real-time monitoring of chiral transformation in chemical and biological systems. We report on the synthesis of naphthalenediimide appended L-glutamate (NDI-L-Glu) that self-assembles into chiral supramolecular structures under physiological conditions. Specifically, NDI-L-Glu shows a mixture of left- and right-handed helices under physiological conditions, and any deviation from the ambient biochemical environment has a remarkable influence on the chirality of these structures. For instance, acidic environments shift the helicity to left-handedness while the alkaline conditions reversed the helical structures to right-handedness, thereby mimicking the molecular virulence mechanism of tobacco mosaic virus (TMV). The chirality of these supramolecular assemblies can also be controllably tuned by using temperature as an external stimulus, allowing reversible flip of helicity.

## Introduction

Chirality is one of the most fascinating natural phenomena, leading to a specific handedness of biological structures, and directing the biochemistry to choose a specific homochirality in life processes^1–3^. Nature is able to mysteriously translate molecular chirality into selective left- or right-handed homochirality at various structural levels of organisms. Tobacco mosaic virus (TMV) provides an exquisite example of chiral self-assembly in natural systems. In TMV the whole virion is a right-handed nucleoprotein helix, composed of a single type of protein subunits closely packed in a regular helical capsid, interpenetrated by a strand of RNA^4^. A remarkable feature of this self-assembled TMV is the ability of the virus to destabilize the capsid structure once inside the host cell, and release the genetic material for subsequent infection. Anionic amino acids, in particular, multiple glutamate (Glu) residues play an important role in disrupting the chirality of the TMV capsid superstructure^5^. Several attempts have been made to design chiral chemical architectures with an effort to mimic the function and geometry of complex biological systems using molecular self-assembly^6–10^. However, none of these systems has been able to mimic the inherent complexity of even the simplest biological organisms, such as TMV.

Nonetheless, there is no doubt that the control of the helicity in man-made supramolecular ensembles is of utmost significance, as it is closely connected with the topic of chirality transmission^11^. This has important mechanistic implications in life sciences. Notably, the biomolecular self-assembly in nature is dominated by ensembles of non-covalent interactions. The inherent dynamic nature of non-covalent interactions, accompanied with their susceptibility to manipulation using physical and chemical stimuli offers an idealistic avenue to mimic the complexity of chirality-directed biological self-assembly^12–16^. To achieve this, various factors such as vortex motion^17^, stirring^18,19^, magnetic field^20^, redox forces^21^, solvophobicity^22^, pH^23–25^ and light^26^ have been used to manipulate the chirality of superstructures^27–32^. The use of extended π-systems provides an additional avenue to direct supramolecular chirality via enhancing non-covalent interactions^3,33–35^.

Herein, we designed and synthesized **NDI-L-Glu** complex combining the π-conjugated naphthalenediimide (NDI) as a core component with the anionic amino acid L-Glu as an appendage. The proposed **NDI-L-Glu** complex mimics the natural supramolecular chiral self-assembly of TMV. The NDI molecule offers a compact, planar and aromatic, π-conjugated system that has found numerous applications in particularly organic electronics due to their excellent n-type conductivity, stability and ability to self-assemble into supramolecular structures^36–40^. On the other hand, the chosen appendage L-Glu offers one of the most fascinating entity to achieve chiral supramolecular assemblies with inherent reversibility. In nature, L-Glu residues of protein play an important role in defining their secondary structure, conformation and overall stability^41^. This is because L-Glu not only has especially high helix-forming propensities; these anionic amino acid residues also bring high conformational entropy to proteins, leading to the possibility of multiple protein structures and their destabilisation in the presence of an appropriate trigger^41^. While the importance of L-Glu residues in chiral self-assembly is reflected from the above-discussed case of TMV. Another well-known example in the human body is sickle cell anaemia that is caused by the replacement of glutamate with valine in the β-globin chain of haemoglobin, leading to protein aggregation^42^. As such, our current work demonstrates that akin to the biological systems, the **NDI-L-Glu** complex is not only able to self-assemble into chiral supramolecular helices under physiological conditions; but also the deviation from the physiological environment leads to reversible chiral transformation in the structure of **NDI-L-Glu** super-helices.

## Results

**Figure 1:**
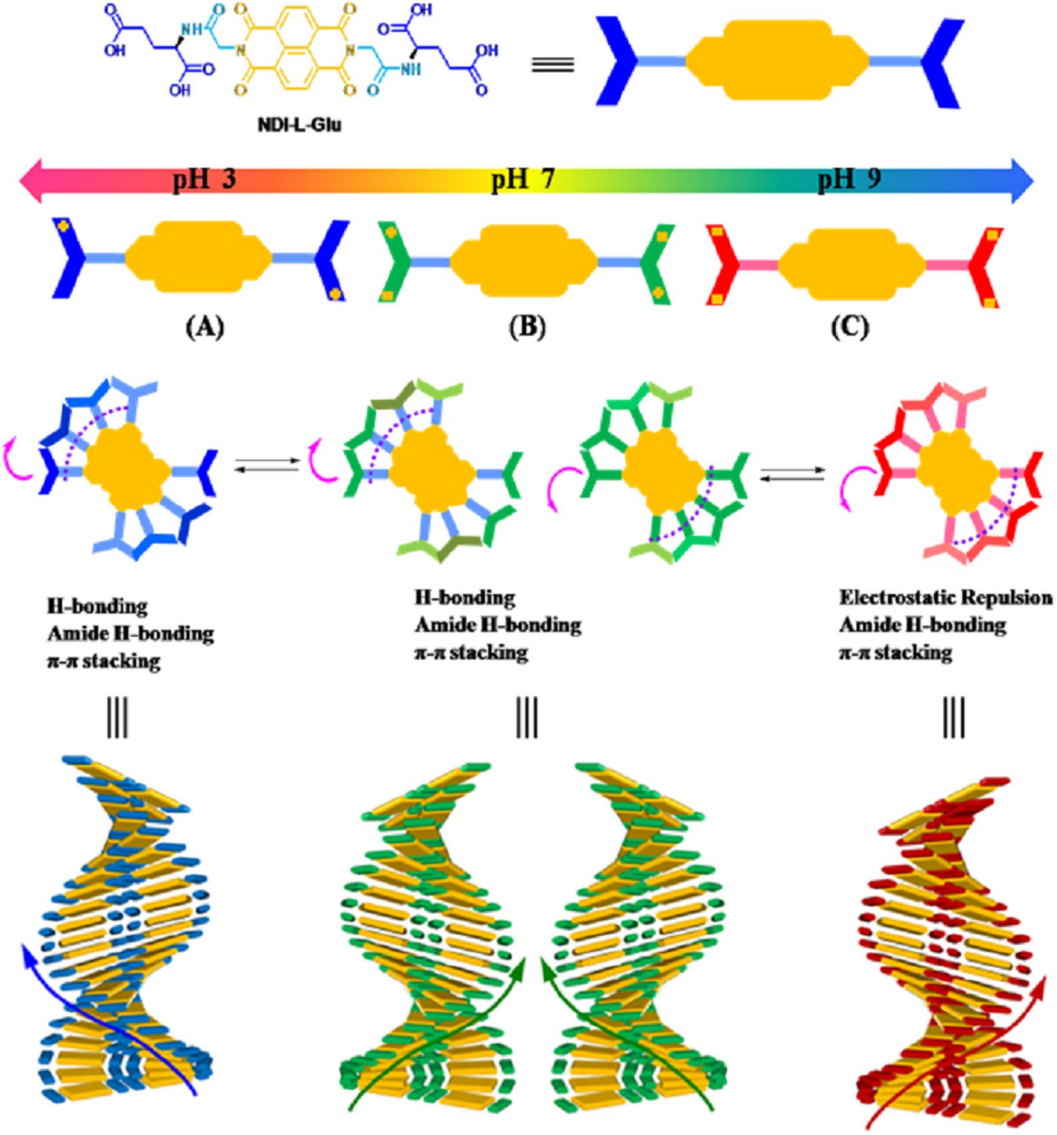
Schematic representation of **NDI-L-Glu (1)** aggregation pathways, showing the stacking of dimers bound using two hydrogen bonds at (**A**) acidic pH resulting in left-handed helix, (**B**) the self-assembly of 1 in the range of physiological pH which is initiated by both left- and right-handed oligomers due to the equilibrium between these two conformations at the molecular level, resulting in both left- and right-handed twisted superstructures, and (**C**) alkaline pH which resulted in right-handed helical superstructures. This morphology dependency of these self-assemblies on pH shows the importance of the hydrogen bonding between two molecules of **1** among other interactions in the self-assembly process. The conformation of **1** changes dihedral angular twist direction resulting in opposite direction helical superstructures.

In this work, we present a supramolecular helical assembly that can be tuned to the opposite helicity by controlling pH and temperature with a small N-substituted NDI molecule for the first time (Fig. 1). In brief, we have synthesised NDI appended chiral L-glutamic acid (**1**; **NDI-L-Glu**) in both imide positions that was fully characterised by means of FTIR, NMR spectroscopy, MALDI-TOF MS and elemental analysis (for detail see Supplementary Information). In this case, **NDI-L-Glu** (**1**) possesses two important features resulting in the formation of helical structures: (i) the NDI core which optimize the dispersive interactions such as π-π stacking and van der Waals interactions between the cores, and (ii) the L-glutamic acid pendants prompting the formation of helical structures through a strong hydrogen-bonded network. Thus, such arrangement prevents crystallization and favours helical growth of structure in particular directions as shown in Fig. 1. The helical supramolecular structures formation was imagined by transmission electron microscopy (TEM) and scanning electron microscopy (SEM). Furthermore, UV-vis absorption, fluorescence, CD, XRD and TD-DFT were used to evaluate the aggregation mode.

### UV-vis absorption and emission investigation

**Figure 2:**
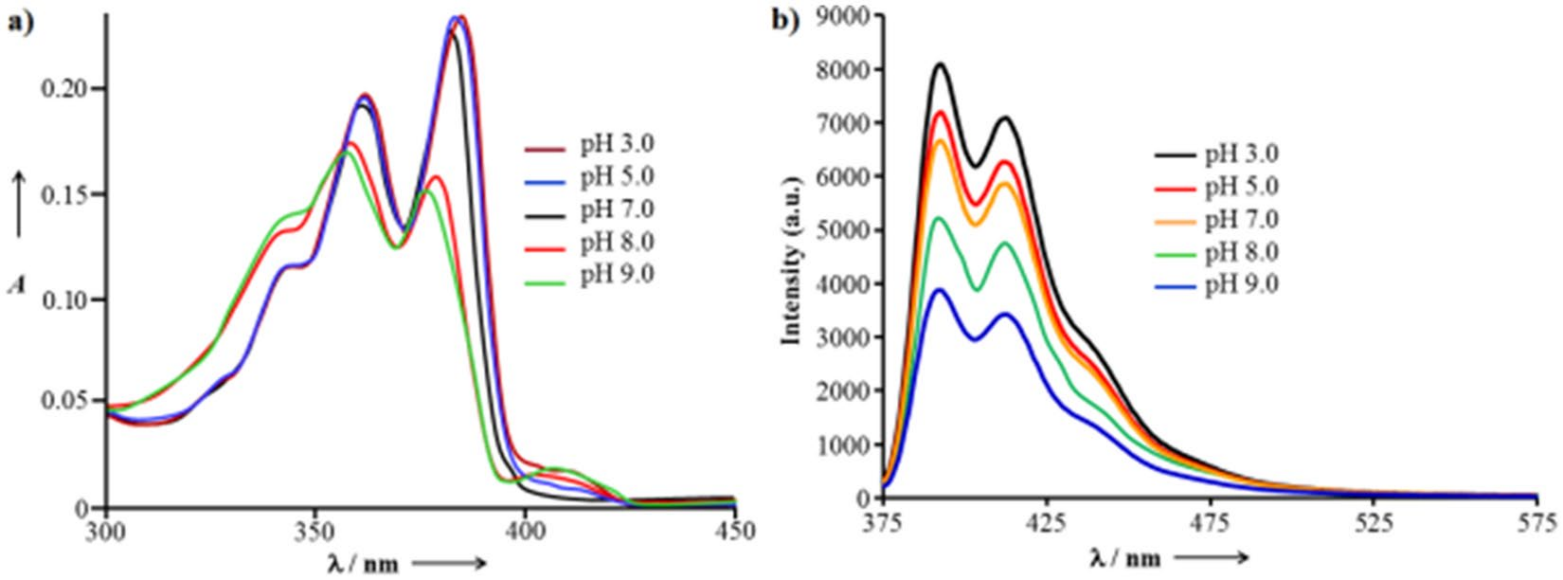
UV-vis absorption and emission spectra of **NDI-L-Glu (1)**. (**a**) The UV spectra and (**b**) fluorescence spectra (λ_ex_ = 360 nm) of 1 (0.5 × 10^−5^ M) at pH between 3 and 9 in Robinson-Britton buffer.

The UV-vis absorption spectra of **NDI-L-Glu** (**1**) (0.5 × 10^−5^ M) in Robinson-Britton buffer showed sharp absorption peaks at 360 nm and 380 nm with a short shoulder at 335 nm at neutral pH (7.0), which is characteristic of the S0 → S1 transition^23^. To gain insight into this phenomenon, pH-induced self-assembly was established by monitoring the UV-vis absorption as a function of the pH value. The λ_max_ of **NDI-L-Glu** (**1**) is red-shifted from 380 nm at pH 7.0 to 385 for **1** + H^+^ at pH 3.0, while blue-shifted absorption was observed to 372 nm for pH 9.0 (Fig. 2a)^43^. Typically, when the pH values of solution were changed from 3.0 to 9.0, a decrease in absorption intensity along with blue-shift absorption and the appearance of shoulder at 425 nm were observed. This clearly demonstrates two different modes of assembly. Fluorescence spectroscopy provides further evidence for pH-dependent aggregation of **NDI-L-Glu** (**1**) (Fig. 2b). Compound **NDI-L-Glu** (**1**) shows emission maxima at 392 and 418 nm (λ_ex_ = 360 nm) in buffer at pH 7.0, which increases with decrease in pH (7.0 to 3.0). However, emission is decreased with increasing the pH from 3.0 to 9.0. This clearly demonstrates the mode of aggregations is completely depending on employed pH. The UV-vis absorption and fluorescence spectroscopy results suggest that the formation of helical structures *via* H-bonding between L-Glu along with face-to-face π-stacks of NDI core comparable to that observed for the J- and H-aggregates in pH 3.0 and pH 9.0, respectively^36^.

We also examined the effect of increase in base percentage (0–50 equiv.) with the addition of NaOH solution (Fig. S2). It was observed that fluorescence emission quenched significantly and saturated at 40 equiv. of NaOH aq. (Fig. S2b). These results clearly demonstrate that the change in pH direct the mode of aggregations. Furthermore, the effect of salt on supramolecular structures was also evaluated by emission spectroscopy using NaCl and Na_2_SO_4_ slats, respectively (Fig. S3a,b) at pH 9. There were no significant emission intensity changes indicating that there is no any role of salt on assembly formation and deformation.

### Circular dichroism (CD) spectrum investigation

**Figure 3:**
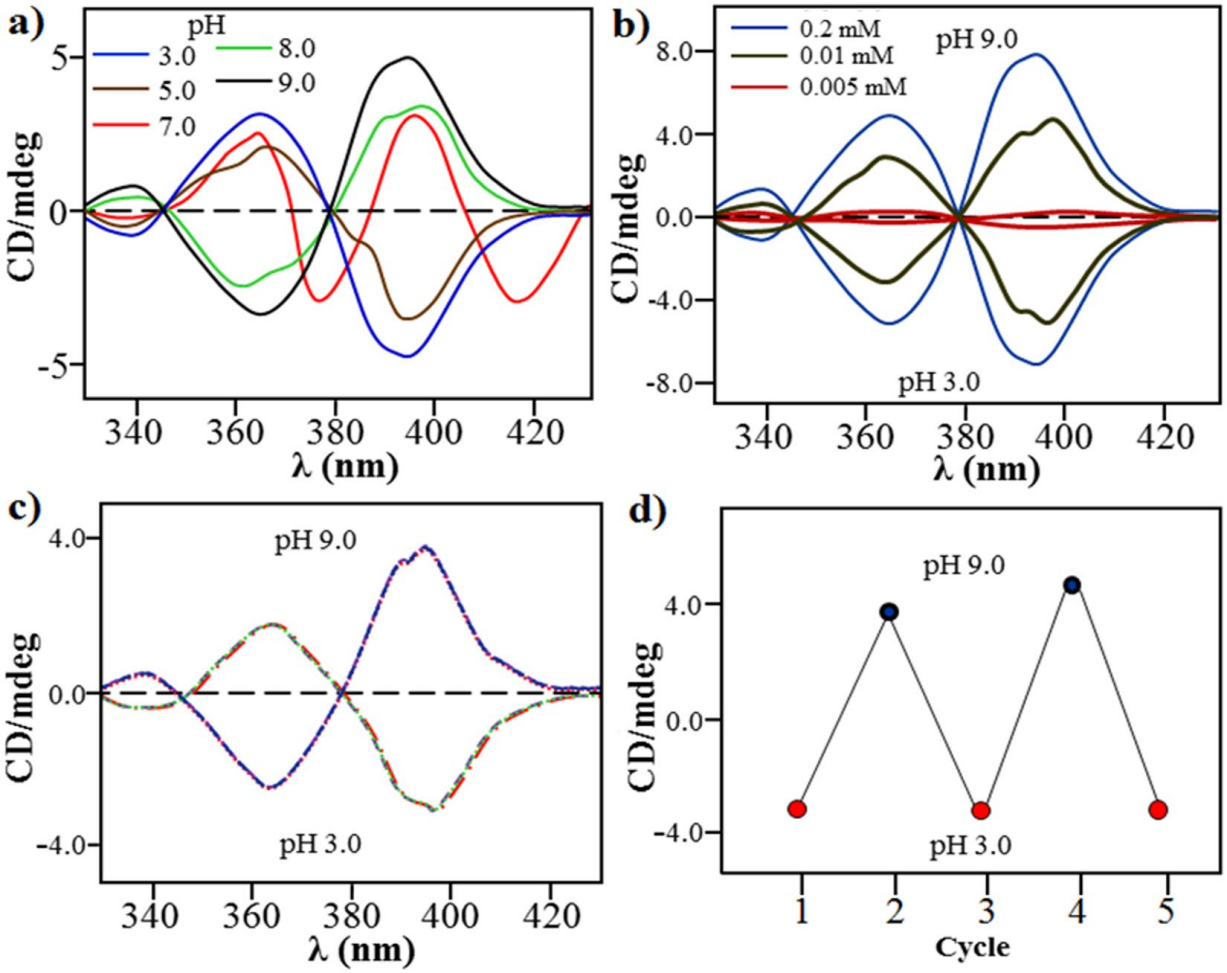
pH-dependent Chirality Inversion. (**a**) Circular dichroism (CD) spectra of **NDI-L-Glu** (**1**) (c = 10^−5^ M) at 3 to 9 pH range, and (**b**) the CD spectra of 0.5, 1.0 and 1.5 mM solution concentrations of **1** at pH 3 and 9. (**c**) CD spectra of **1** solution with cyclic switching between pH 3 and 9, and (**d**) changes in the CD signal intensity at 362 nm of **NDI-L-Glu** (**1**) in Robinson-Britton buffer solution upon pH changes between acidic (pH = 3.0) and alkaline (pH = 9.0) conditions, respectively.

Circular dichroism(CD) spectrum of **NDI-L-Glu** (**1**)in buffer at pH 7.0 at 20 °C gives two positive bands at 364 and 395 nm with zero-crossing of the Cotton effect at 370, 385 and 405 nm along with the appearance of two positive Cotton effect features at 378 nm and 416 nm, revealing a racemic mixture of the self-assembly (Fig. 3a)^44^. However, at higher pH (9.0 and 8.0), two typical CD signals are observed i.e. negative cotton effect at 362 nm and positive cotton effect at 385 nm with zero-crossing of the Cotton effect at 379 nm. Interestingly, when the solution pH is changed from 9.0 to 3.0, the Cotton effect is completely reversed i.e. positive Cotton effect at 362 nm and negative cotton effect at 387 nm with zero-crossing of the Cotton effect at 379 nm, which confirms that these chiral forms had opposite Cotton effects. The matching zero-crossing of the Cotton effect at 379 nm with a completely opposite CD signal as the pH is changed from 9.0 to 3.0, confirms a reversal of the chirality within the self-assembly. The changes in the UV-vis absorption and fluorescence spectra and the CD Cotton effect take place at the same pH value, which confirms the pH-induced helix reversal.

To gain further insight, we chose two pH values, i.e. 9.0 and 3.0. It can be clearly seen that CD signals increase at both the pH, albeit in right- and left-handed helical structures, respectively (Fig. 3b) as the concentration of **1** is increased. Typically, the CD spectrum of **NDI-L-Glu** (**1**) shows two negative signals at 362 nm and a positive signal at 385 nm with a zero-crossing at 379 nm, which corresponds to the π-π* transition of the NDI moieties at pH 9.0. However, a completely opposite and reversal of the CD spectrum is observed at pH 3.0 as shown in Fig. 3c.

Remarkably, pH cyclic phenomenon with the supramolecular chirality was reversible (see inset Fig. 3d), such that when the pH of **NDI-L-Glu** (**1**) in Robinson-Britton buffer was adjusted to pH 9.0, the positive Cotton effect signal appeared at 362 nm, but the signal reversed on acidification to pH = 3.0. This CD spectral change on changing the pH can be repeated several times without any significant change in the intensity of the CD signal in both acidic and basic pH (Fig. 3d). Linear dichroism (LD) spectroscopy experiments guarantee that this reversed Cotton effect is not caused by convection-induced alignment as illustrated in ESI Fig. S3^19,21^. LD spectrum shows a small response, which increases after stirring. The LD spectrum clearly indicates that the length of a helical structure. This is most likely associated with the alignment of the **NDI-L-Glu** (**1**) helical fibrous aggregates upon stirring the long helical assemblies are well-dispersed in solution. These results clearly suggest that a certain fibre length is required to induce the LD and CD effects.

**Figure 4:**
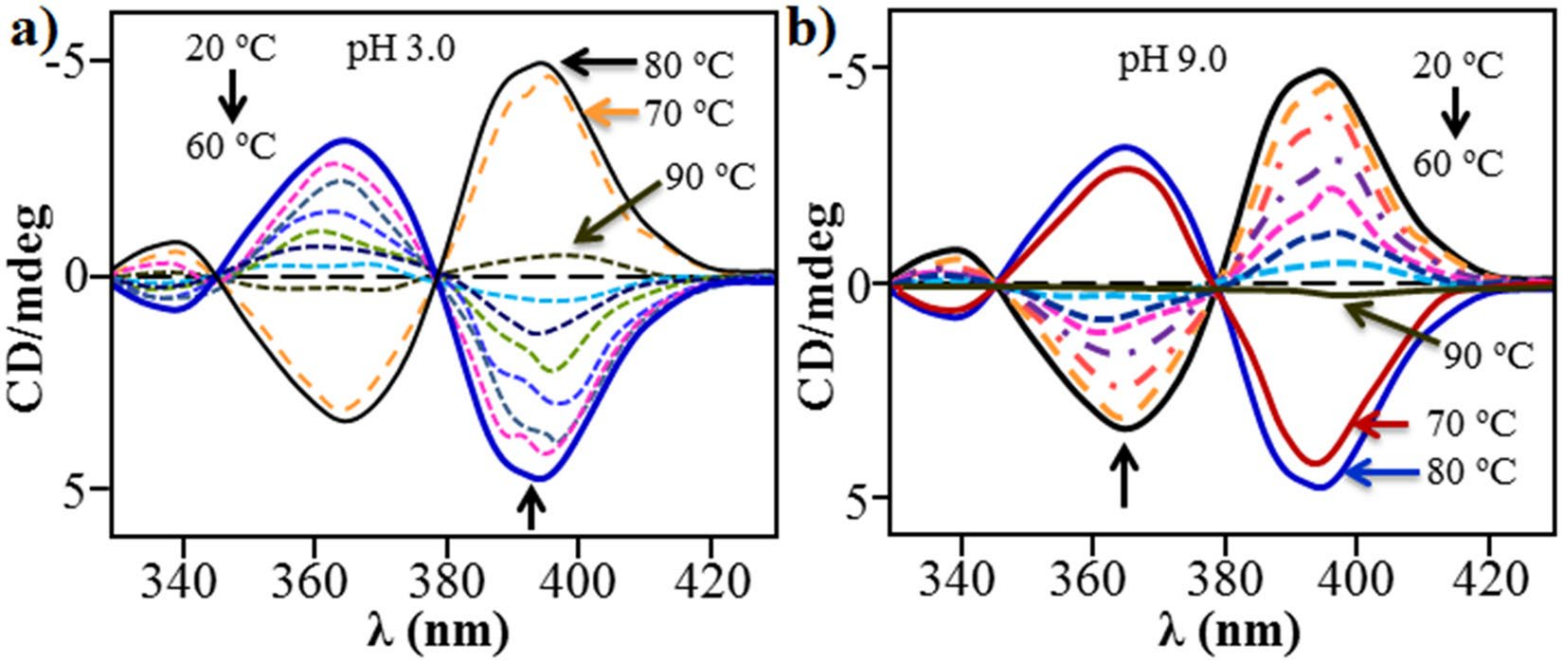
Temperature-directed chirality inversion. The temperature dependency of CD signals with spectra taken between 20 and 90 °C at (**a**) pH 3, and (**b**) pH 9. These temperature-dependent CD measurements clearly shows diminishing of CD signal at 60 °C and reversing the CD at >70 °C irrespective of the pH.

The self-assembly of **NDI-L-Glu** (**1**) was further analysed by monitoring the changes in the CD spectroscopic data as a function of the temperature at both the pH i.e. 3.0 (Fig. 4a) and 9.0 (Fig. 4b). The **NDI-L-Glu** (**1**) at pH 3.0 prefers a left-handed helix at 20 °C, and with increasing temperature the CD signatures continuously decrease to an extent that they become inactive at about 60 °C. Very interestingly, the **NDI-L-Glu** (**1**) assemblies are flipped into a right-handed helix when the temperature is subsequently increased to 70 °C and a further enhancement of CD signals is observed at 80 °C (Fig. 4a). Similarly, **NDI-L-Glu** (**1**) at pH 9.0 reveals a completely opposite reversal signal i.e. right-handed helix reversal to left-handed helix at ~70 °C (Fig. 4b). As expected, under both acidic and alkaline conditions (pH 9.0 and 3.0), CD signal diminishes when heated at 90 °C, indicating dis-assembly of supramolecular structures.

Thus, CD studies provide strong evidence that the helical handedness (left to right) of the assembly could be modulated reversibly by adjustment of pH values from pH 3 to pH 9, respectively. Transformation between left to right-hand helical structures can also be realised by temperature control, i.e. at pH 3.0 and 20 °C compound **1** produced left hand helical structure which can be tuned to right handed helical structure at 70 °C and vice-versa. Similarly, at pH 9.0 at 20 °C, **1** assembled into right-handed helical structure which reversed to left-handed helix at 70 °C. This phenomenon can be explained with the fact that with compound **1** bearing acid side chain, at the near-physiological pH compound remains as azwitterion; while in the acidic medium, protons interact with the amino group of glutamic acid, prompting the formation of an H-bonding network that further leads to a left handed helix. On the other hand, in a pH range of 7–9, the induced helical sense flips into right-handed conformation due to the break of the original H-bonding network as illustrated in Fig. 1. This phenomenon is similar to that by Tanaka *et al*.^24^, wherein the natural insulin fibrils were observed to reverse by the adjustment of the pH values. Aida^25^ group also reported an achiral, non-neutral amino acid bearing basic side chains which showed a similar phenomenon. Notably, while the reversal in the chirality of the TMV helicity is not known once the virion enters the host cell and faces a change in the pH conditions, but it is known that the Glu residues play an important role in the disintegration of the viral capsid. Our findings suggest a likelihood of such phenomenon in biological systems, and may prompt further investigations in this area.

To gain further understanding in controlled self-assembled microstructures by pH, TEM and SEM were carried out.

### Transmission electron microscopy investigation

**Figure 5:**
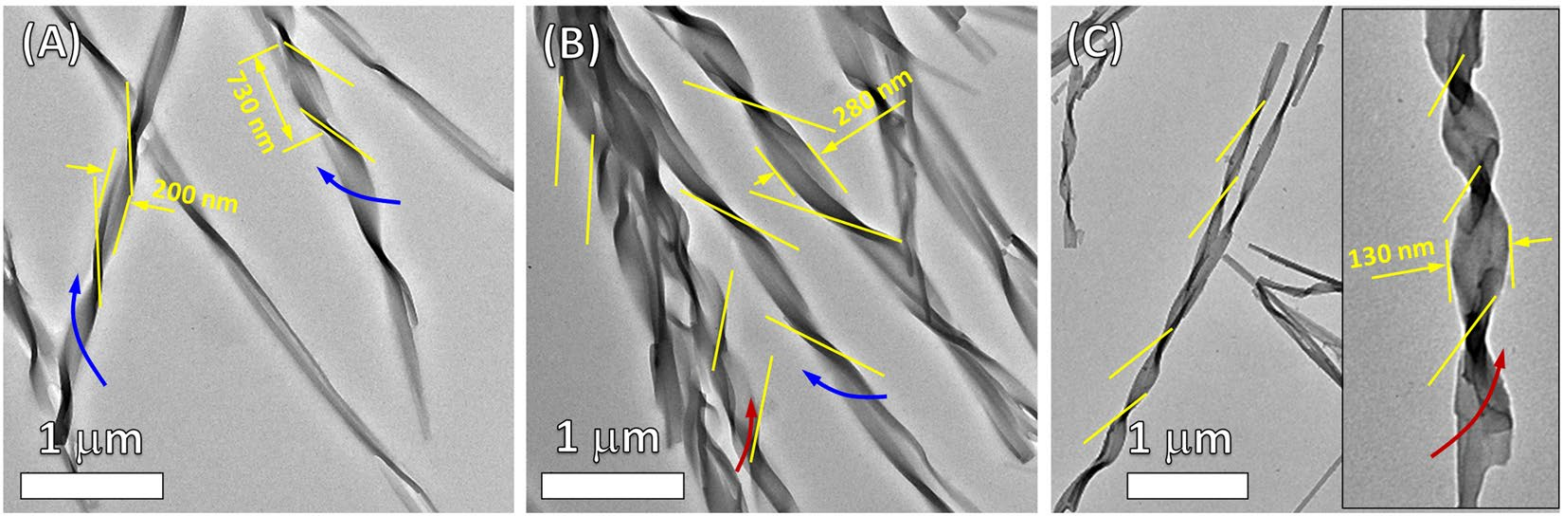
Visualisation of various pH dependent self-assembly by TEM analysis. TEM micrographs of the helical supramolecular microstructures of **NDI-L-Glu (1)** produced by solvent evaporation at pH (**A**) 3.0 (left handed twisted ribbons), (**B**) 7.0 (a mixture of left and right handed twisted ribbons), and (**C**) 9.0 (right handed twisted ribbons).

TEM measurements of the self-assembled microstructures of **NDI-L-Glu** (**1**) were performed by solvent evaporation of **1** at various pH values on holey carbon grids as shown in Fig. 5. It can be clearly seen in Fig. 5A that the formation of left-handed twisted ribbon with approximately 200 nm in width and a twist half period of 730 nm was obtained from supramolecular assembly of **NDI-L-Glu** (**1**) in the solution with pH 3. Interestingly, when the solution pH 7.0 (neutral) was used, the **NDI-L-Glu** (**1**) self-assembled into a mixture of left- and right-handed twisted ribbon (Fig. 5B). Under alkaline condition (pH 9, Fig. 5C), the **NDI-L-Glu** (**1**) assembled into right-handed twisted ribbons with the width of less than 130 nm and the twist half period of approximately 1 µm. These TEM observations are in good agreement with the data obtained from the CD spectroscopy (Fig. 3a).

### Scanning electron microscopy investigation

**Figure 6:**
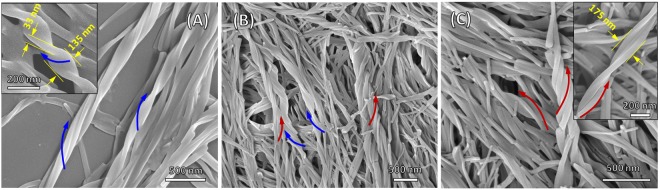
SEM microscopy employed to visualise helical pH dependent self-assembly. SEM micrographs of the helical supramolecular microstructures of **NDI-L-Glu (1)** produced by solvent evaporation at pH (**A**) 3.0 (left handed twisted ribbons), (**B**) 7.0 (a mixture of left and right handed twisted ribbons), and (**C**) 9.0 (right handed twisted ribbons).

To further examine the hierarchical twisted microstructures from the self-assembly of the compound **NDI-L-Glu** (**1**) in solutions of different pH values, the self-assembled supramolecular structures were observed under SEM (Fig. 6). The SEM micrographs were recorded by solvent evaporation of **NDI-L-Glu** (**1**) on silicon wafers. Figure 6 shows almost similar features as illustrated in above TEM images (Fig. 5). At the solution pH of 3 (Fig. 6A), the self-assembly of **NDI-L-Glu** (**1**) resulted in highly preferred well-organized left-handed twisted ribbon with approximately 33 nm in thickness, 135 nm in width and hundreds of nm in twisted haft periods. The width of ribbon at pH 3 in SEM image is larger than that observed under TEM, which may be ascribed to the role of substrate in influencing the self-assembly of compound **NDI-L-Glu** (**1**) due to potential interaction of **NDI-L-Glu** (**1**) with substrates during self-assembly (carbon grid for TEM and silicon wafer for SEM). A mixture of both right- and left-handed twisted ribbons was also observed in SEM micrograph at the solution pH 7 and the supramolecular assemblies rich in right-handed twisted ribbons were obtained under alkaline condition (pH 9).

### Density functional theory investigation

**Figure 7:**
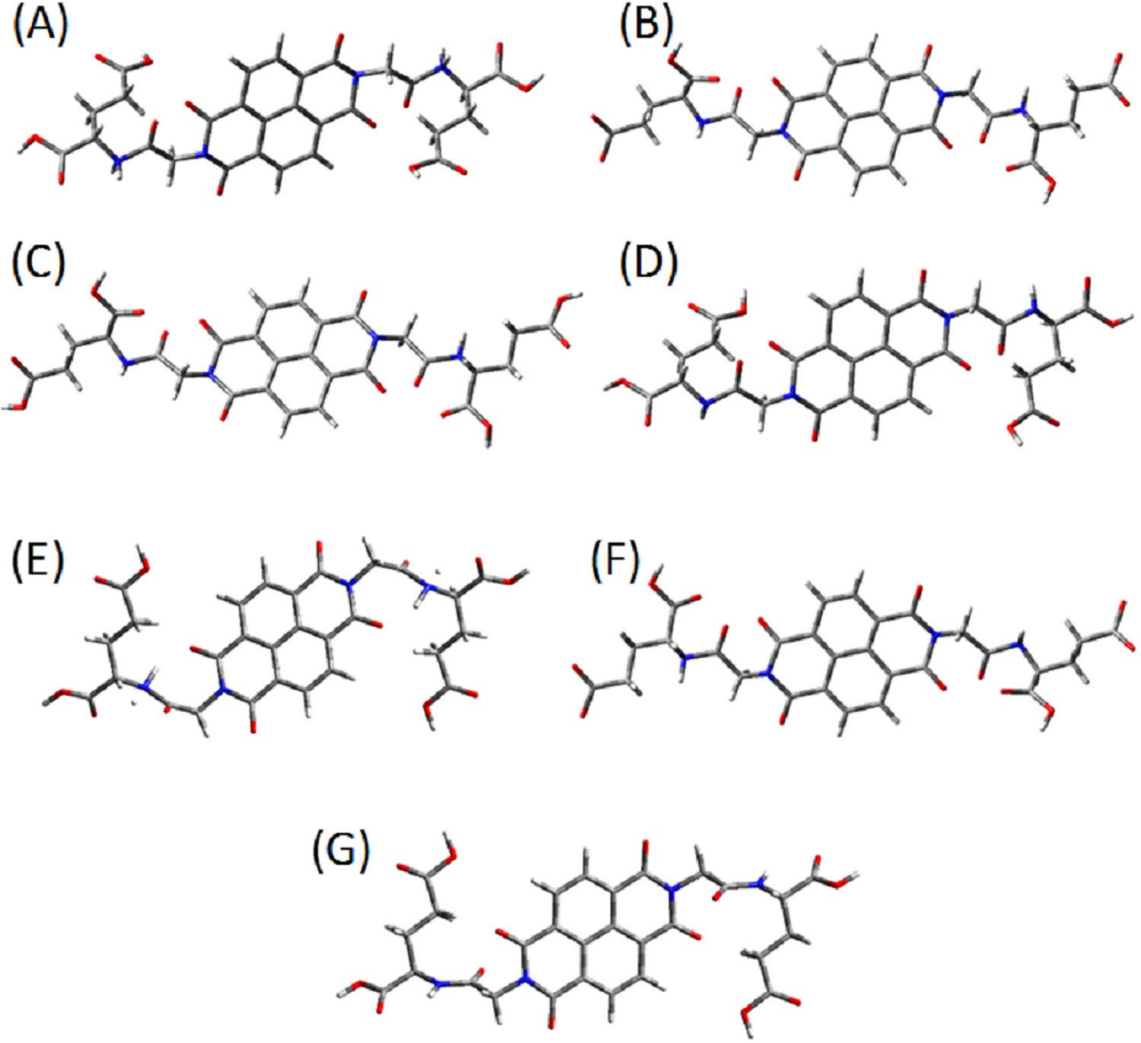
The initial molecular conformations of positively charged **NDI-L-Glu** (**1**) dication (**A**), negatively charged dianion (**B**), and neutral conformations (**C** and **D**) considered for geometrical optimization. The optimized (**E**) dication, (**F**) dianion and (**G**) neutral NDI-L-glu molecular conformations as calculated using DFT B3LYP/3–21 G level of theory.

The CD spectra of **NDI-L-Glu (1)** were produced by molecular modelling at neutral, dianion and dication structures simulating various pH conditions. *In vacuo* Time Dependant Density Functional Theory (TD DFT) calculations were conducted using the Gaussian 16 suite of programs^45^ and the B3LYP/6–311+G(d,p) level of theory after geometry optimizing the molecular structures using DFT B3LYP/3–21G level of theory. The initial conformation chosen for the dication, where the amine groups are protonated, puts the glutamic acid moiety in a position to maximize the charge interaction with positive charge centred on the amine (See ESI Fig. 7A). Conversely, in the initial chosen conformation for the dianion, the farthest carboxylic group of the glutamic acid moiety is deprotonated, which puts the centres of the negative charges the farthest apart to minimize internal charge repulsion (Fig. 7B). For the neutral molecule, these two conformations were adopted to be geometrically minimized (Fig. 7C,D). Our TD-DFT calculations revealed that the dianion and dication species keep the same general conformations after geometry optimization, while the two initial conformations of the neutral species both converge to a structure similar to the one representing the dication species (Fig. 7E–G).

**Figure 8:**
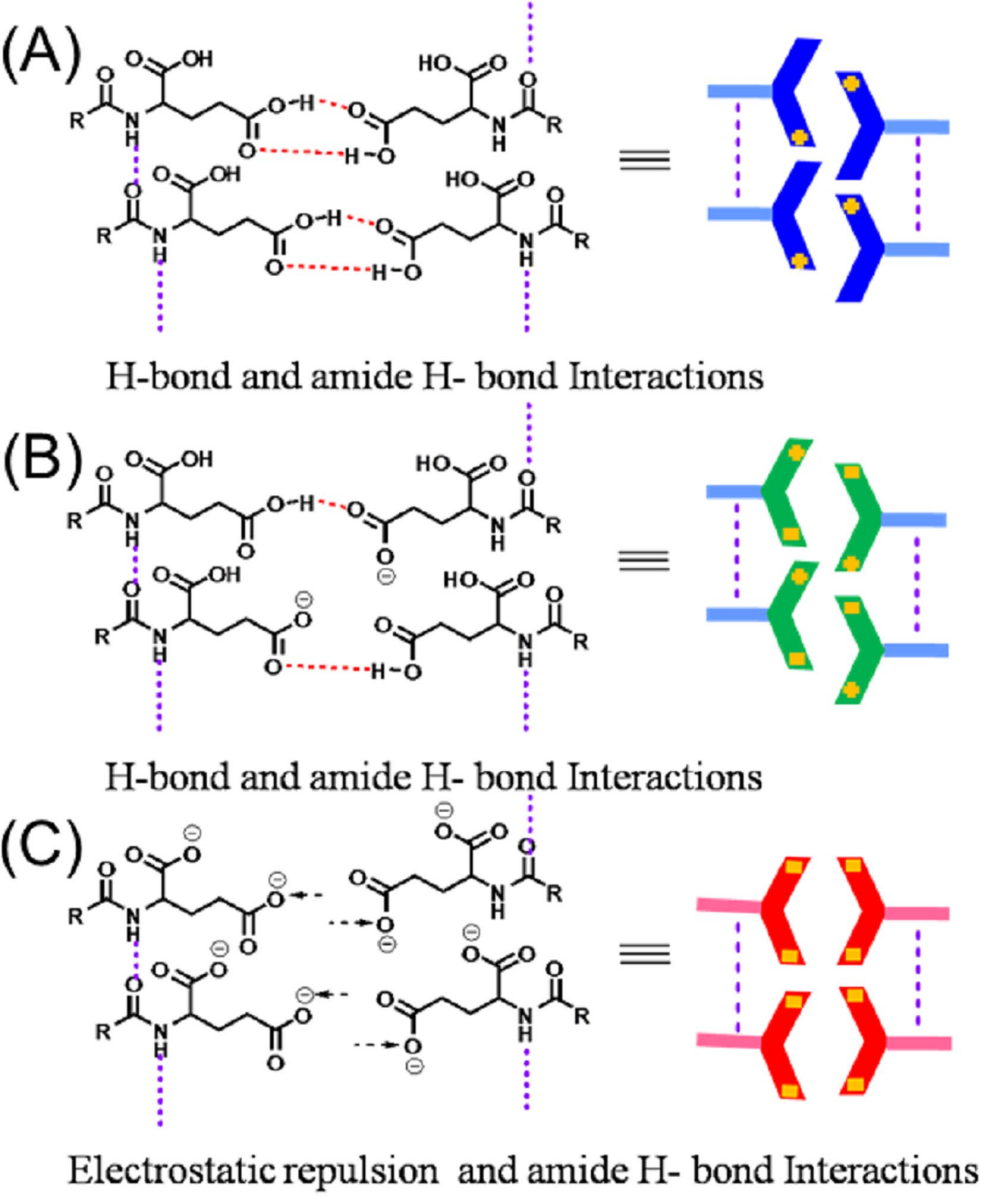
Schematic representation of the pH responsive supramolecular polymer formation of **NDI-L-Glu** (**1**); (**A**) at pH 3; amide H-bonding and expected π-π staking interactions; (**B**) at pH 7; H-bonding, amide H-bonding and expected π-π staking interactions; (**C**) at pH 9; electrostatic repulsion, amide H-bonding and expected π-π staking interactions.

Further, GaussSum software^46^ was used to obtain the circular dichroism spectra of these four species by processing the B3LYP/6–311+G(d,p) calculations output files. The model CD spectrum of dianion shows a positive signal for the outer shell transition, which is in agreement with CD signal under alkaline conditions. An opposite negative CD signal for outer shell transitions of the dication of **NDI-L-Glu** (**1**) was obtained from modelling, in agreement with the experimental results of supramolecular assembly under acidic conditions. The **NDI-L-Glu (1)** with no carried charge converged to very similar conformation after geometry optimization but TD DFT gave opposite CD signals for these two very similar conformations (see ESI Fig. S4). This suggests that the CD signal is very sensitive to molecular conformation. In comparison, experimental results shown in Fig. 3a for pH 7 gave a different pattern, which may result from a combined CD signals of various conformations of neutral **1** species especially with possibilities of zwitterion formation and equilibrium of protonated, deprotonated and neutral species at the neutral pH as shown in Fig. 8.

### X-ray diffraction (XRD) investigation

Figure S5 shows the XRD pattern of **NDI-L-Glu** (**1**) crystalline nature when deposited from buffer at neutral pH (7.0). However, when aggregates deposited from basic pH 9.0 mixture become partly amorphous nature and similar at acidic pH (3.0) lower crystallinity was observed as compared to pH 7.0 and 9.0, respectively. These results consistent with observations in SEM and TEM analysis (Figs 5 and 6).

### Dynamic light scattering (DLS) investigation

To get detail insight on size of self-assembled aggregates of **NDI-L-Glu** (**1**) at various pH such as 3, 7 and 9, we conducted DLS experiments and the results are depicted in Fig. S6. It is clearly observed that at pH 3, 7 and 9, the sizes of self-assembled aggregates are about 471 nm, 912 nm and 216 nm, respectively. We presume that the smaller size (216 nm) of **NDI-L-Glu** (**1**) aggregates at pH 9 is due to the electrostatic repulsion contribution. These results are correlated with the smaller pitch (250 nm) of right handed helix (p-helix) at pH 9.0. Whereas at pH 3, the size of **NDI-L-Glu** (**1**) aggregates is larger (471 nm) resembles to the large pitch size (730 nm) of left handed helix.

## Discussion

In summary, Fig. 1 illustrates the prospective model conformation of **NDI-L-Glu** (**1**) carrying positive and negative charge and in its zero net charge states self-assembling to give supramolecular structures based on the results of CD, TEM, SEM, XRD and DLS analysis. The arrows show the directional orientation of **NDI-L-Glu** (**1**) in self-assembly relative to the neighbouring molecules. The absence of charges at neutral pH results in less internal strain in the molecule making both conformations equally possible, giving both left and right-handed twist direction superstructures. The hydrogen bonding involved in the self-assembly process is affected by the geometrical conformation of the two carboxylic acid functional groups in the L-glutamic acid moieties appended to the NDI core. The pH variation changes the conformation of this region of **1** molecule and thus affecting direction of interaction between molecules in the self-assembled supramolecular structure. In a highly acidic environment, the **NDI-L-Glu** (**1**) amide group may be protonated^47^ where the molecule can accommodate a positive charge, while under alkaline conditions the carboxylic group may be deprotonated resulting in negative charges in the N-substituted moieties as illustrated in Fig. 1. The molecule will carry a zero net charge at the pH of the isoelectric point. The charge distribution in the molecule induced by the pH of the environment affects the conformation of the molecule and therefore the shape of the supramolecular self-assemblies. We believe clustering of similar charged molecular ions must be associated with counter ions in the same assembly and may involve solvent molecules to moderate the repulsive charge interaction in the molecular self-assembly and maintain universal charge balance.

**Figure 9:**
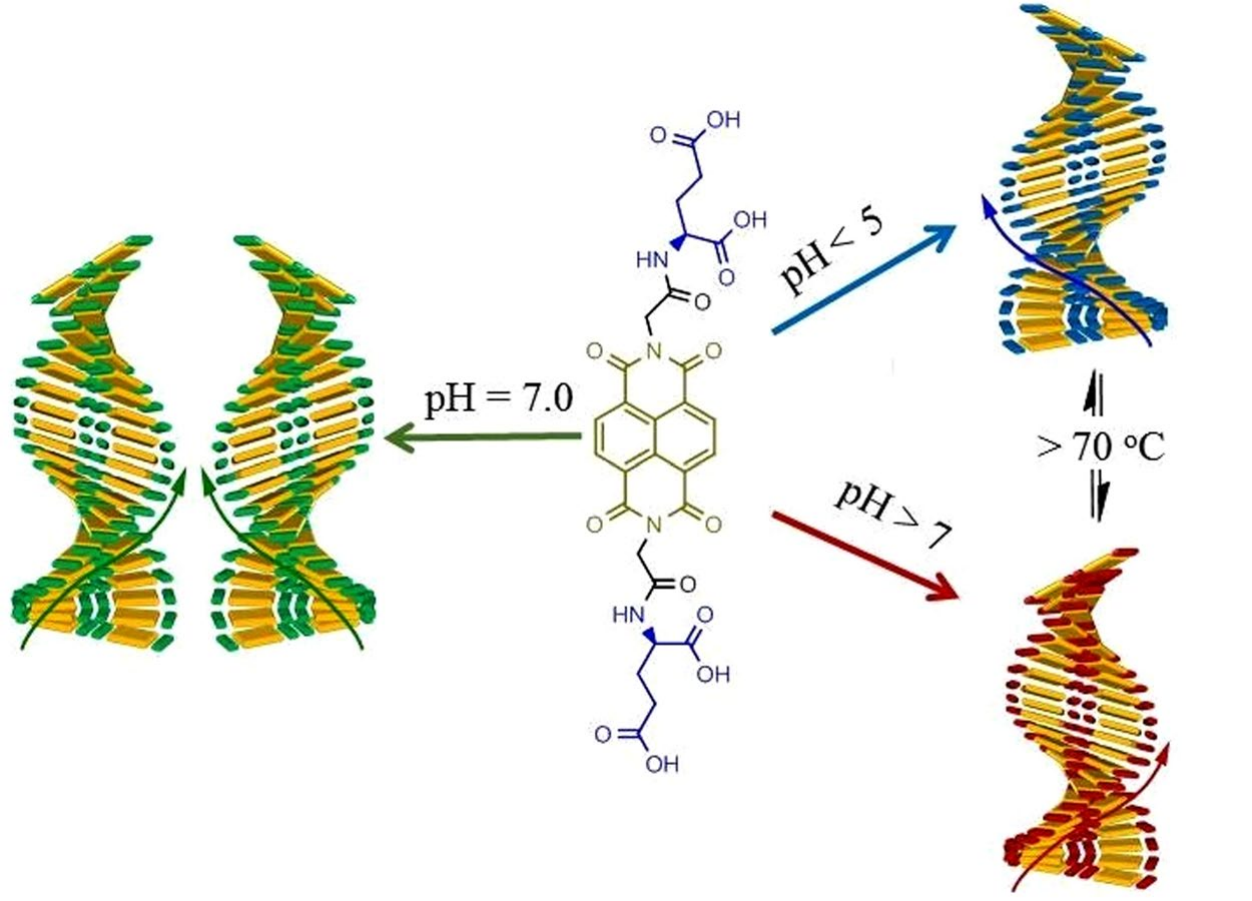
Graphical illustration of the handedness of a man-made supramolecular object can be controllably tuned with the action of pH and temperature.

On the other hand, that the **NDI-L-Glu** assemblies are flipped into a right-handed helix by the increase of temperature. It may be due to electrostatic interactions become to overcome the effect of the H-bonds by the increase of temperature. It is a significant finding that the handedness of a man-made supramolecular object can be controllably tuned with the action of pH and temperature (Fig. 9). As such, we demonstrated the reversible supramolecular assembly displaying a pH- and temperature-dependent switching of the optical activity. Further, the induction of optical activity with the supramolecular self-assembly was reversible across multiple cycles without losing activity. Similarly, temperature-controlled switching of macroscopic chiral supramolecular materials may offer switchable electronic properties, for example, for controlling conductivity and charge carrier mobility in the presence of different biological analytes for real-time sensing of biological events.

To the best of our knowledge, the multistimuli-directed supramolecular helical chiral nanostructures inversion triggered by both pH and temperaturein an aqueous medium. As per our knowledge this is the first example to demonstrates preference for one type helical structures controlled by both pH and temperature and vice-versa. The supramolecular system studied here offers not only the more in-depth experimental understanding of chirality, but also provides a unique way to control the helical assemblies by changing the pH and temperature stimuli. In addition, the present system offers a new and simple approach to the design of new chiral supramolecular polymers, which can provide a good illustration of chirality control with right and left handed preference by either controlling pH or temperature. These tunable chiral assemblies may find applications in challenging areas, such as, purifying racemic mixtures of commercially important drugs, detecting subtle changes in the spatio-temporatal configuration of biomolecules during disease progression, obtaining more in-depth mechanistic understanding of host-pathogen interactions, and engineering environmentally-responsive macroscopic chiral objects. These new findings will have high impact to a wide range of disciplines across supramolecular chemistry, chirality, biochemistry, molecular pathogenesis, nanomedicine, and engineering.

## Materials and Methods

### General Methods & Materials

Naphthalene dianhydride, L-glutamic acid, D-glutamic acid, glycine, dry DMF, EDC, Et_3_N and DIPEA were purchased from Sigma-Aldrich, Bengaluru, Karnataka, India and were used as received. Ethanol (AR grade 99.9%) was purchased from S.D. fine chemicals Limited (SDFCL), India. Robinson-Britton buffer was prepared by using standard protocol (*Annali di Chimica*, **1974**, 64, 409–412). There action solvents were degassed for 10–15 min using nitrogen gas. The reactions were carried out under nitrogen atmosphere. Silica-gel chromatography technique was used to purify the synthesised compounds. ^1^H and ^13^C NMR spectroscopy spectra were measured on Bruker Avance-500 MHz and 125 MHz spectrometer at 27 °C. Tetramethylsilane was used as an internal standard. All experiments were performed in deuterated chloroform (CDCl_3_), CDCl_3_+ deuterated TFA and DMSO-*d*_6_ wherever required. Electron spray ionization method was employed to ionize the samples with a spray voltage. Mass spectrometry measurements were performed using Fourier transform based high resolution mass spectrometry. IR-Spectra were recorded using Thermo Nicolet Nexus 670 spectrometer in the form of non-hygroscopic KBr pellets.

#### Synthesis

Target derivative **NDI-L-Glu** (**1**) was synthesized in three steps according to the procedures given in the accompanying Supplementary Information.

#### pH Experiments

A stock solution of **NDI-L-Glu** (10^−4^ M) was prepared in Robinson-Britton buffer solution. A aliquot (0.2 mL) of the stock solution was diluted in 1 mL with varying pH of buffer (p H = 9.0, 8.0, 7.0, 5.0 and 3.0) and acidic pH was adjusted by the addition of HCl (0.1 M) in buffer while the basic pH by addition of NaOH solution (0.1 M). The mixture was allowed to stand overnight and was then subjected to spectroscopic measurements. The pH of the aqueous layer was monitored using a pH meter.

#### pH cycle measurements

A 0.2 mL stock solution was added to basic buffer at pH 9.0 or pH 3.0, and the mixture was stirred at room temperature for 2 h, further was allowed to stand overnight and was subjected to spectroscopic measurements.

### Spectroscopic measurements

#### UV–Vis measurements

UV–vis absorption spectra were recorded on a Cary-50, spectrometer using 1 cm path length cuvette. A 0.2 mL aliquot of the stock solution of **NDI-L-Glu** (conc. = 10^−3^ M) was transferred to Robinson-Britton buffer, and made up to 2 mL volume. The entire mixture was allowed to equilibrate for 2 h prior to the spectroscopic measurements.

#### Fluorescence Measurements

Emission spectra were recorded on a Horiba Jobin Yvon FluoroMax®-4–Spectrofluorometer which is equipped with an injector port and stirrer at 20 °C. All the measurements being performed in a quartz cuvette with a 1 cm path length (λ_ex_ = 360 nm).

#### Circular Dichroism

CD spectra were recorded on an AVIV 202 CD spectrometer under a nitrogen atmosphere. The measurements were performed in a quartz cuvette with a 1 mm path length over the range of 330–430 nm in Robinson-Britton buffer solution, similar to UV-vis absorption.

#### SEM imaging

First the silicon wafer was washed by acetone, ethanol followed by Milli Q water. SEM samples were prepared by solvent evaporation on a silicon wafer and then sputter coated with gold for 10 s at 0.016 mA Ar plasma (SPI, West Chester, USA) for SEM imaging using a FEI Nova NanoSEM (Hillsboro, USA) operating at a high vacuum which provided direct visualisation of the self-assembled aggregated structures.

#### Transmission Electron Microscopy (TEM) imaging

The samples were prepared by evaporating sample solvents on a holey carbon grid and the images were taken on a JEOL 1010 100 kV TEM.

#### LD measurements

LD was measured in a 32 mm bore 20 T Duplex Bitter magnet using a standard polarization modulation technique^48^. Different lines of a Spectra Physics Argon ion laser were used.

#### Molecular modelling

Density functional theory (DFT) calculations with no consideration of dispersion interactions in gas phase was conducted using Gaussian 09 suite of programs.

## Electronic supplementary material

Supplementary Information
