# Supplementary material for: Dynamic multistimuli-responsive reversible chiral transformation in supramolecular helices
Source: Sci Rep. 2018 Jul 25;8:11220. doi: 10.1038/s41598-018-29152-9 (PMC6060148; doi:10.1038/s41598-018-29152-9)
Supplement: Supplementary file 1 — Supplementary Information [file 41598_2018_29152_MOESM1_ESM.docx]

**Supplementary Information**

**Dynamic multistimuli-responsive reversible chiral transformation in supramolecular helices**

Santosh Goskulwad,^1^ Duong Duc La,^2^ Mohammad Al Kobaisi,^2^ Sidhanath V. Bhosale,*^1^ Vipul Bansal*,^2,3^Ajayan Vinu,^4^ Katsuhiko Ariga,^5,6^ and Sheshanath V. Bhosale*^7^

^1^Polymers and Functional Materials Division, Academy of Scientific and Innovative Research (AcSIR), CSIR-Indian Institute of Chemical Technology, Hyderabad 500007, Telangana, India. ^2^School of Science, RMIT University, GPO Box 2476, Melbourne, VIC3001, Australia.^3^Ian Potter Nano BioSensing Facility and Nano Biotechnology Research Laboratory, RMIT University, GPO Box 2476, Melbourne, VIC 3001, Australia.^4^Global Innovative Centre for Advanced Nanomaterials, Faculty of Natural Built Environment and Engineering, University of Newcastle, Callaghan, Newcastle 2308, NSW, Australia. ^5^WPI-MANA, National Institute for Materials Science (NIMS), 1-1 Namiki, Tsukuba, Ibaraki 305-0044, Japan. ^6^Department of Advanced Materials Science, Graduate School of Frontier Sciences, The University of Tokyo, 5-1-5 Kahiwanoha, Kashiwa, Chiba 277-8561, Japan. ^7^Department of Chemistry, Goa University, Taleigao Plateau, Goa-403206, India

**Correspondence and requests for materials** for research proposes should be addressed to S.V.B. (GU) svbhosale@unigoa.ac.in; VB (RMIT):vipul.bansal@rmit.edu.au; SVB (IICT): bhosale@iict.res.in


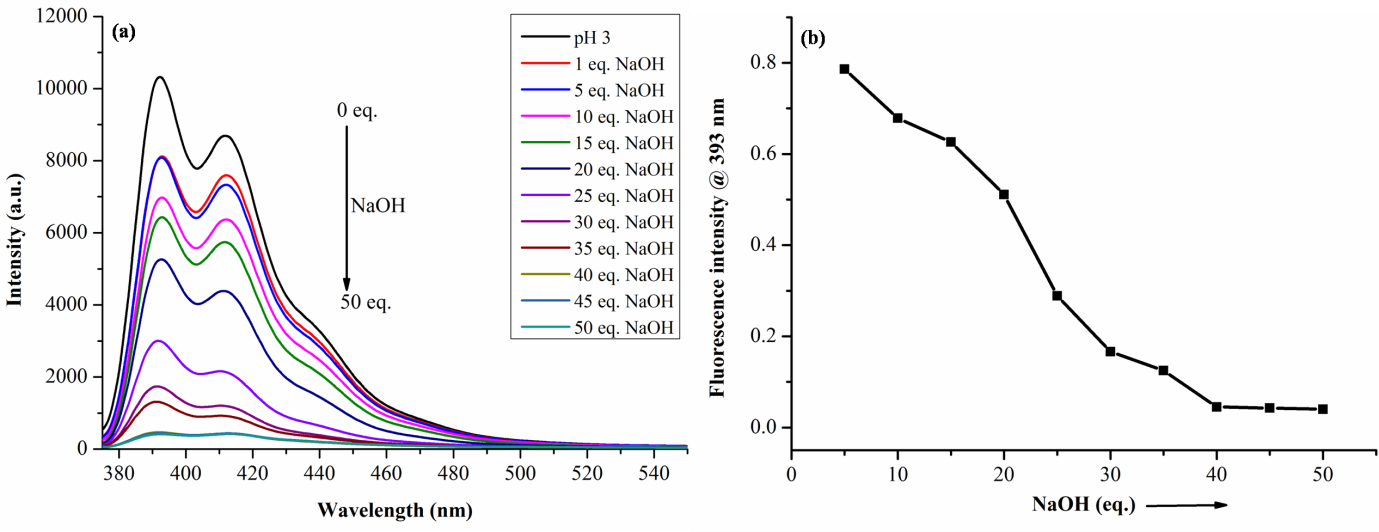


**Figure S1. (a)** Fluorescence emission spectra of **NDI-L-Glu** (**1**) upon addition of NaOH (0- 50 equiv.); (b) Equivalent of NaOH *vs* fluorescence intensity change at 393 nm.


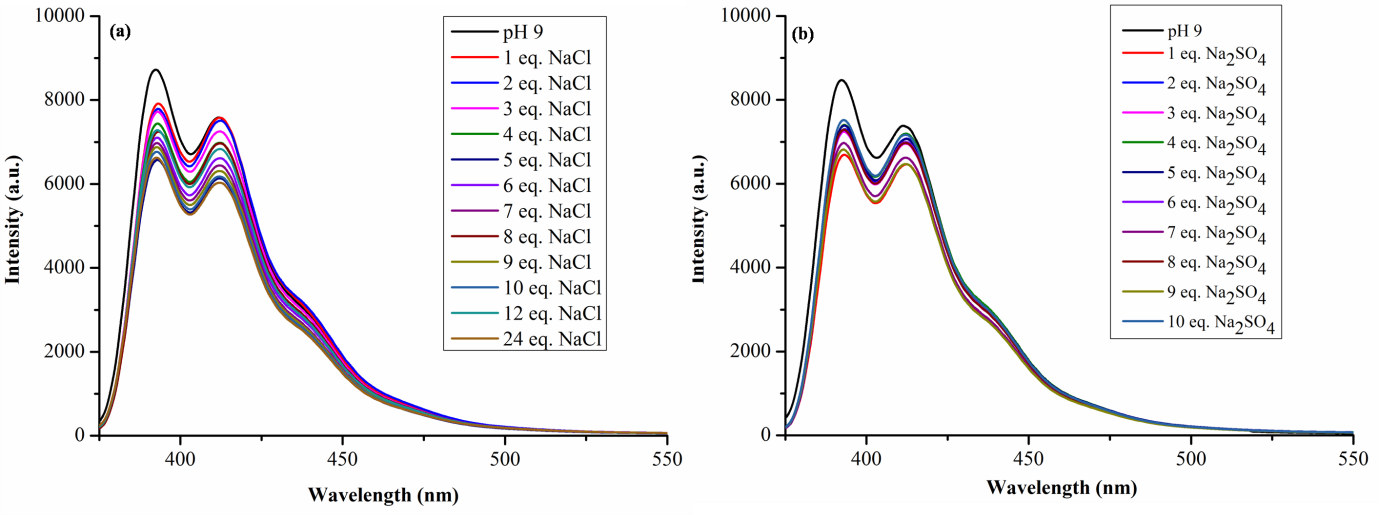


**Figure S2.** Fluorescence emission spectra of **NDI-L-Glu** (**1**) upon addition of salt **(a)** NaCl (0- 24 equiv.) and **(b)** Na_2_SO_4_ (0- 24 equiv.)


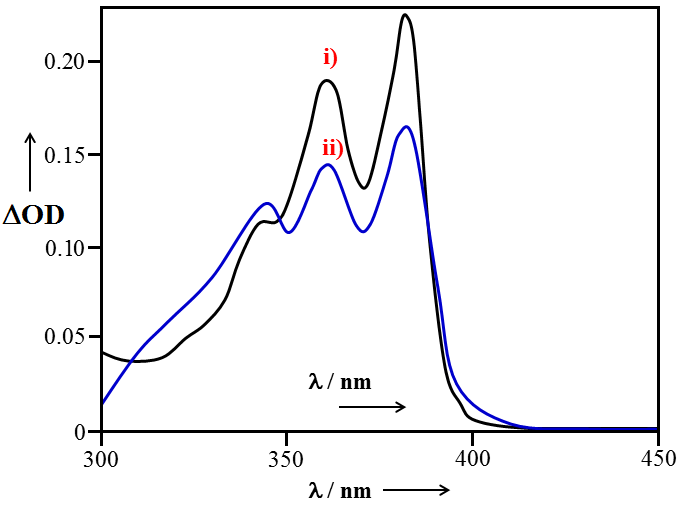


**Figure S3.** LD spectra of **NDI-L-Glu** (**1**) (1 cm cuvette, c = 10^-5^ M) in buffer after cooling (20 ^o^C) without stirring (blue: ii) and after stirring (black: i).

**Figure S4.** The calculated CD spectra of geometrically optimized **NDI-L-Glu** (**1**) (A) dication, (B) dianion, and (C, D) two neutral conformations as obtained at TD DFT B3LYP/6-311+G(d,p) level of theory.


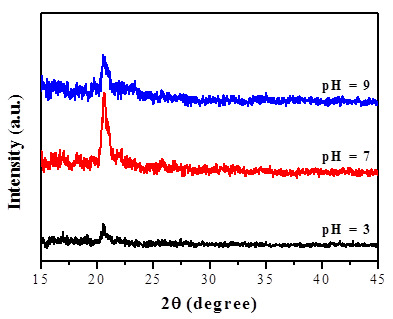


**Figure S5.** XRD of **NDI-L-Glu** (**1**) at various pH.


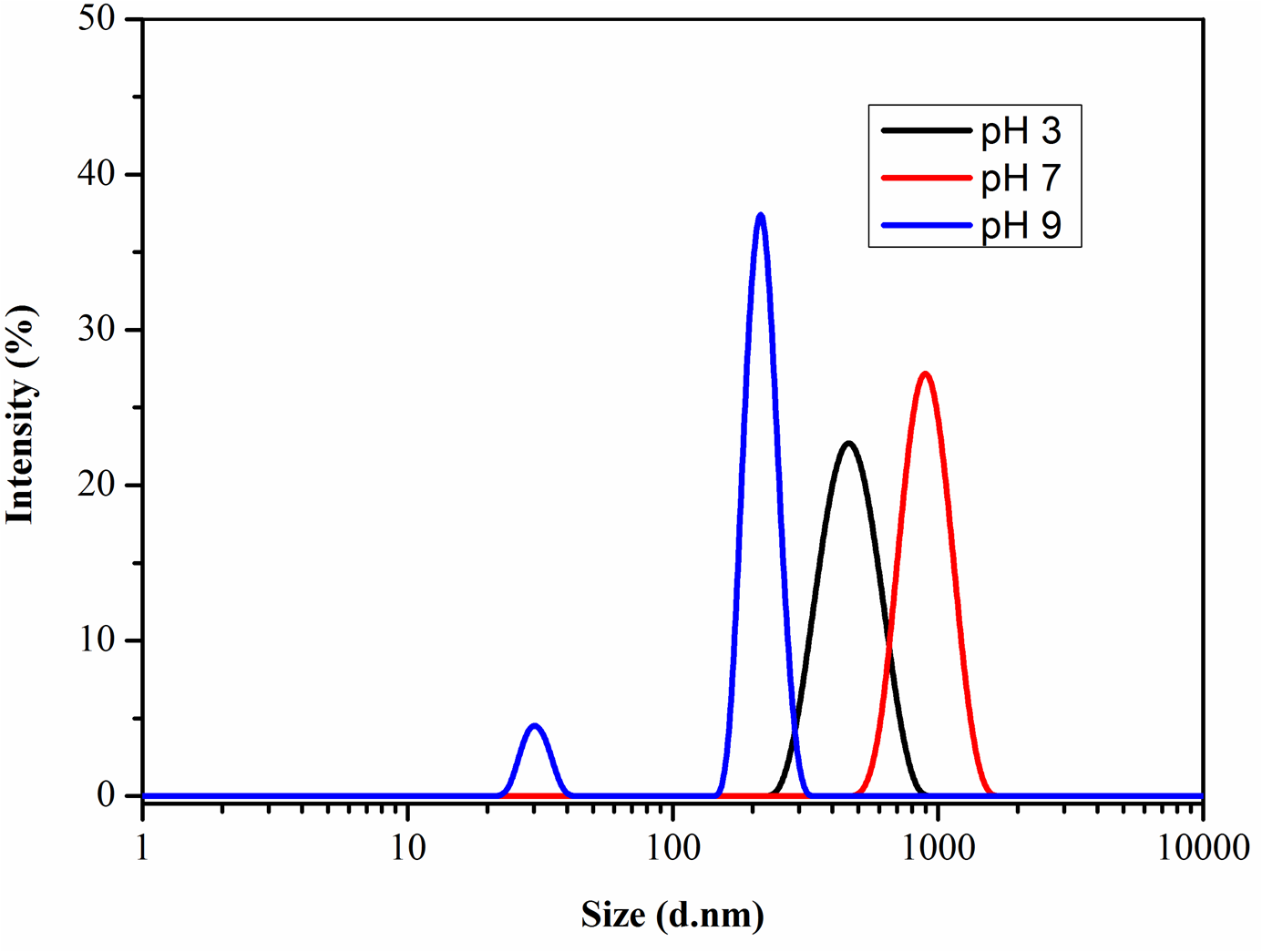


**Figure S6.** Dynamic light scattering (DLS) measurements of **NDI-L-Glu** (**1**); at pH 3, 7 and 9.

***Synthesis of NDI-G1***

**Scheme S1** Synthesis of **NDI-L-Glu** (**1**).

***Synthesis of compound A***

1,4,5,8-Naphthalenetetracarboxylicdianhydride (0.5g, 1.8 mmol) and L-glycine (0.277 g. 3.7 mmol) were suspended in20 mL of dry DMF and the reaction mixture was allowed to reflux for 24 h. After cooling the reaction mixture to room temperature, the precipitate was filtered, washed with water and recrystallized from ethanol, yields compound **A**. ^1^H NMR (300 MHz, DMSO-*d_6_*) δ**:** 8.74 (4H, s), 4.77 (4 H, s).^13^C NMR (75 MHz, DMSO-*d_6_*)δ: 168, 162, 130, 125, 45.HRMS: calculated for C_18_H_10_N_2_O_8_= 382.0437, found = 382.0458.

***Synthesis of compound C***

To a 50 mL round bottom flask compound **A** (100 mg, 0.26 mmol), N-(3-dimethylaminopropyl)- *N*′-ethylcarbodiimide hydrochloride (150 mg, 0.78 mmol) and1-hydroxybenzotriazole hydrate (113 mg, 0.78 mmol ) were added to a 10 mL dry DMF under nitrogen atmosphere. The reaction mixture was stirred for half an hour at 0 ^°^C. Glutamic dimethyl ester (**B**) (166 mg, 0.78 mmol) and DIPEA (0.2 mL) were added to the reaction mixture. The resulting reaction mixture was stirred for 12h at room temperature. After completion of reaction (monitored by TLC) solvent was removed by rotary evaporator under reduced pressure. The reaction mixture was eluted with dichloromethane (50 mL) and the resultant organic layer was washed with aq. NaHCO_3_ (20 mL) and brine (20 mL) dried over anhydrous sodium sulphate and reduced in vacuo. The residue was purified by silica gel column chromatography eluting with 3% methanol: dichloromethane solution to afford compound **C** as yellow color solid (120 mg, 66% yield). M.P. 282-284 °C. ^1^H NMR (500 MHz, CDCl_3_+ deuterated TFA) δ**:** 8.80 (4H, s), 7.71-7.70 (d, *J* =7.62 Hz,2H), 5.09-5.02 (m, 4H), 4.77-4.74 (m, 2H), 3.83 (s, 6H), 3.7(s, 6H), 2.57-2.54 (t, *J* = 7.61 Hz, 4H), 2.31-2.35 (m, 2H), 2.14-2.08 (m, 2H); ^13^C NMR (125 MHz, CDCl_3_+ deuterated TFA) δ: 175.53, 172.72, 162.99, 131.72, 126.90, 126.30, 53.52, 52.79, 52.51, 43.02, 30.06, 26.69; IR (KBr, v cm^-1^):3295, 3083, 3010, 2958, 2850, 1735, 1714, 1669, 1555,1452,1372, 1346, 1247, 1221, 1123, 1009, 985, 88, 778, 670; ESI-MS (*m/z* %): 697 [M+H]^+^ HRMS: calculated for C_32_H_33_N_4_O_14_= 697.1987, found [M+H]^+^ = 697.1998.

***Synthesis of compound* NDI-L-Glu** (**1**)

A solution of compound **C**(100mg, 0.14 mmol) was added to a THF: MeOH (2 mL: 1 mL) solvent mixture. To this solution LiOH (16 mg, 0.16 mmol) in 1 mL of H_2_O was added. The reaction mixture was stirred for 10 h at rt. The completion of reaction was monitored by TLC. The solvent was evaporated under vacuo. To this 10 mL water was added. Then pH of the solution was adjusted to 1 by 1N HCl, which in turn resulted into precipitation of the compound. The precipitate was collected as brown colored solid, **NDI-L-Glu** (**1**) (72 mg, 79% yield). M.P>300 °C. ^1^H NMR (300 MHz, DMSO- d_6_) δ: 8.72(s, 4H), 8.62-8.60 (d, *J* = 7.97 Hz, 2H), 4.74 (s, 4H), 4.31-4.24 (m, 2H), 2.32-2.27 (t, *J =* 7.42 Hz, 4H), 2.00-1.95 (m, 2H), 1.80-1.73 (m, 2H); ^13^C NMR (75 MHz, DMSO-d_6_) δ: 173.59, 172.87, 166.36, 162.32, 130.64, 126.17, 51.17, 42.48, 29.84, 26.47. IR (KBr, *v* cm^-1^): 3297, 3081, 1984, 1716, 1671, 1554, 1453, 1402, 1321, 1246, 1187, 1005, 887, 809, 776, 724.ESI-MS (*m/z* %):639 [M-H]^+^.; HRMS: calculated for C_28_H_23_O_14_N_4_ = 639.1205, found 639.1273.

**
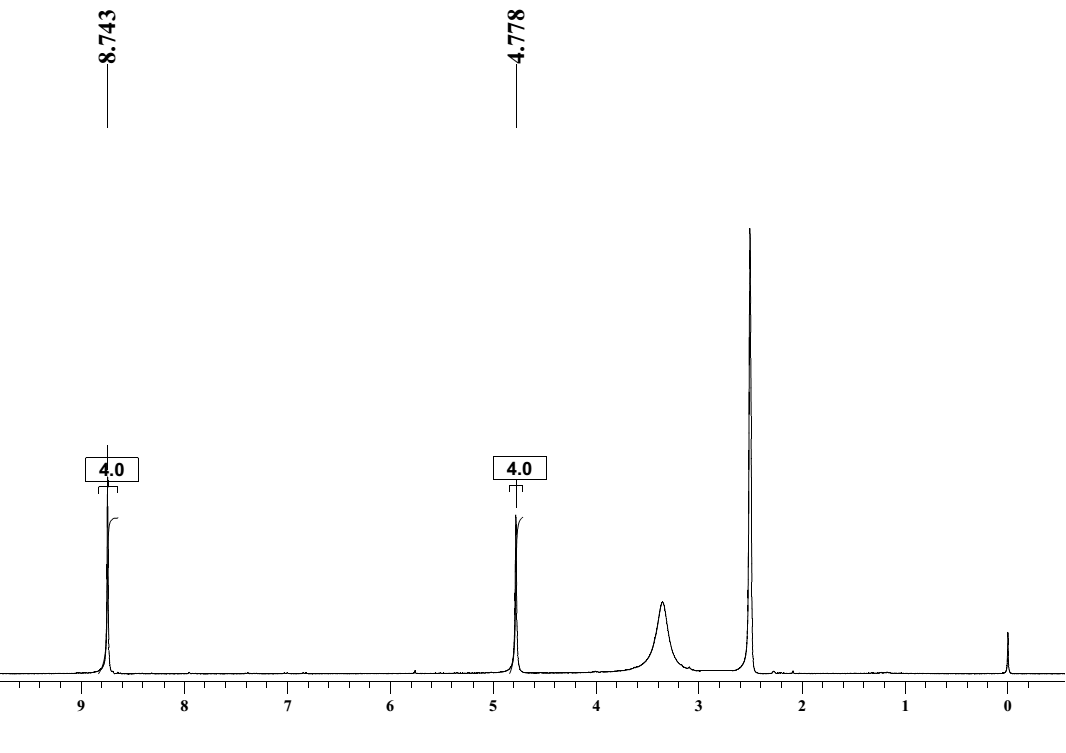
**

**Figure S7.** ^1^H NMR of compound **A.**


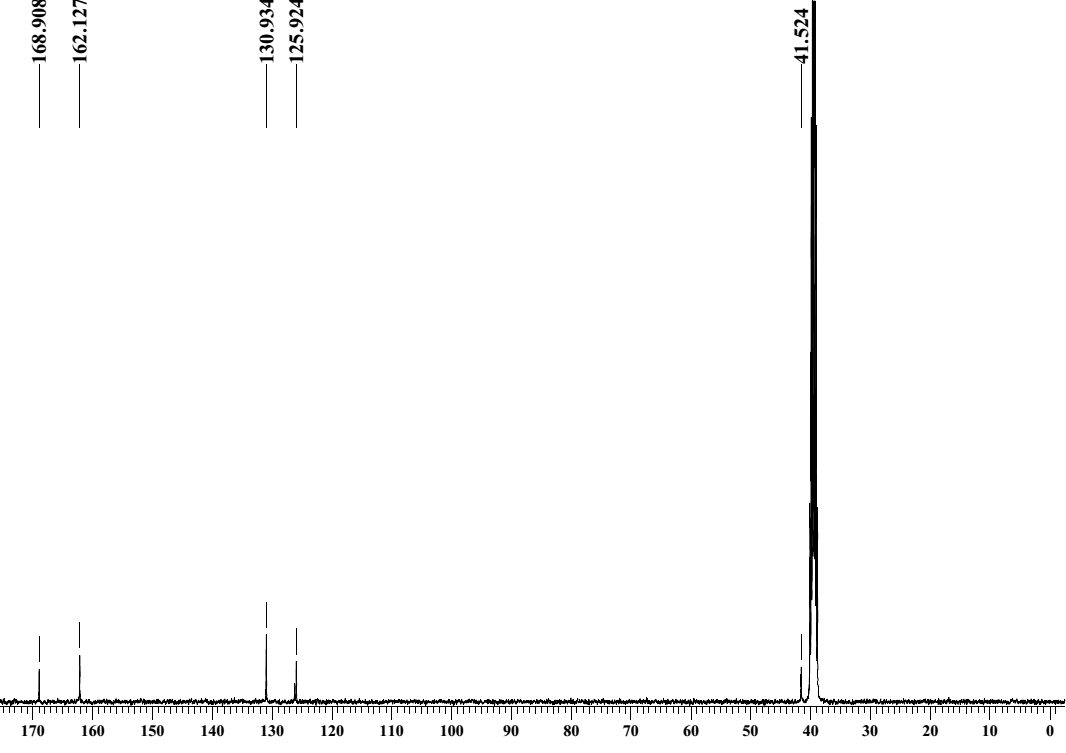


**Figure S8.** ^13^C NMR spectra of compound **A.**


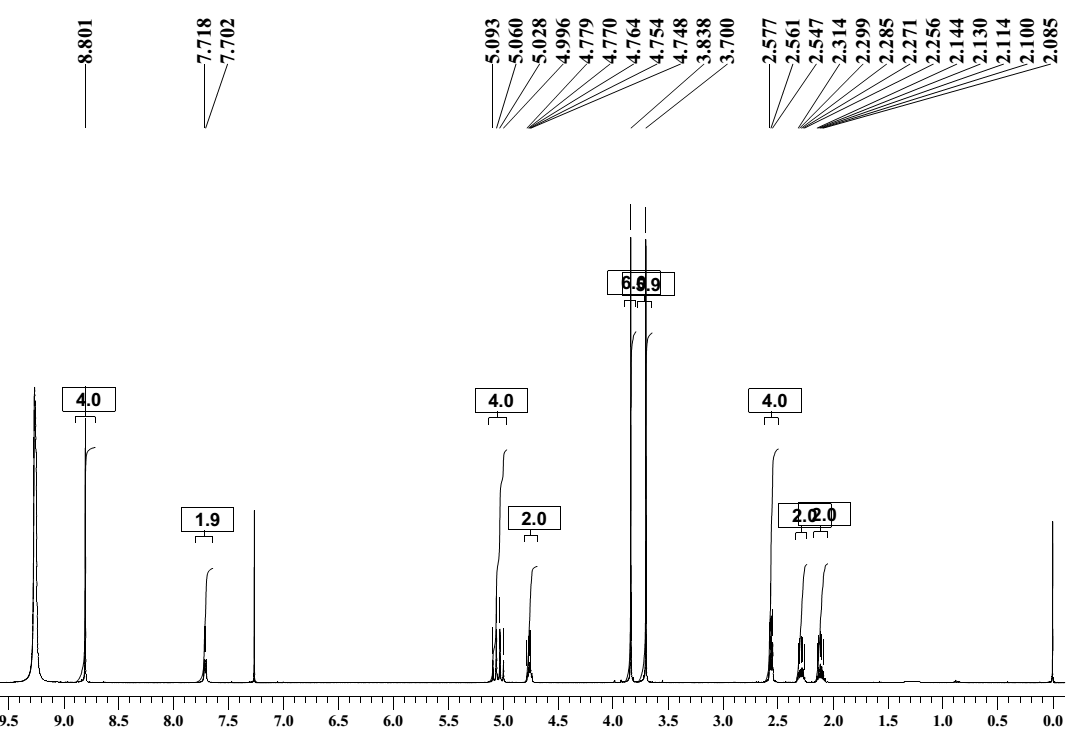


**Figure S9.** ^1^H NMR of compound **C.**

**
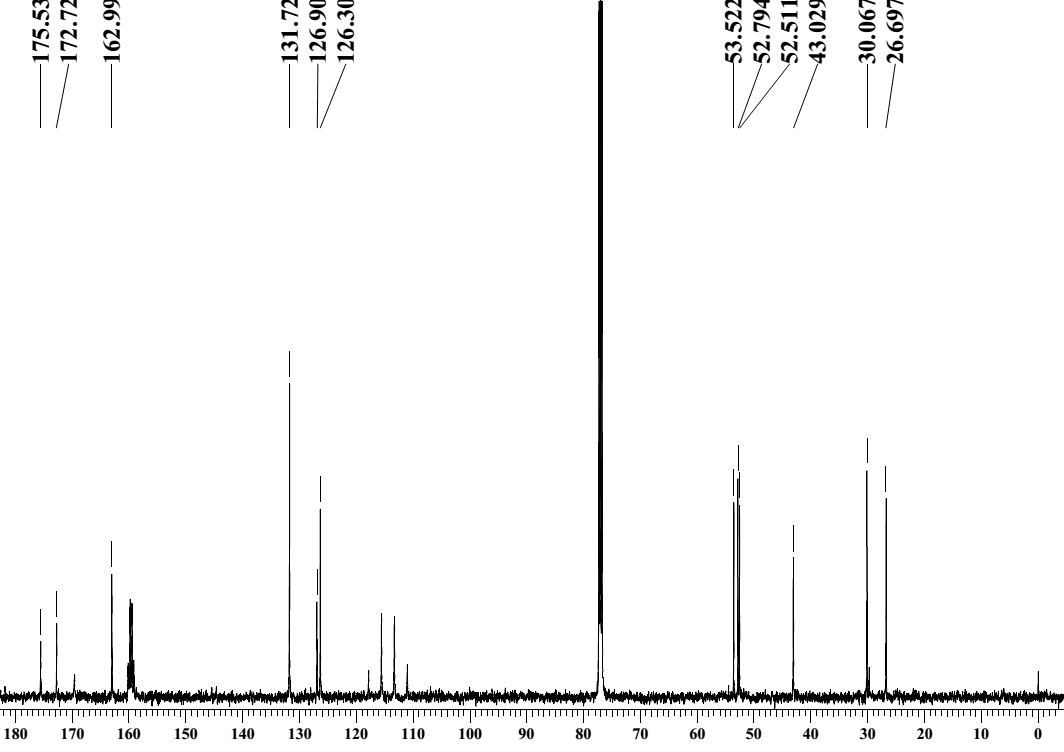
**

**Figure S10.** ^13^C NMR of compound **C.**

**
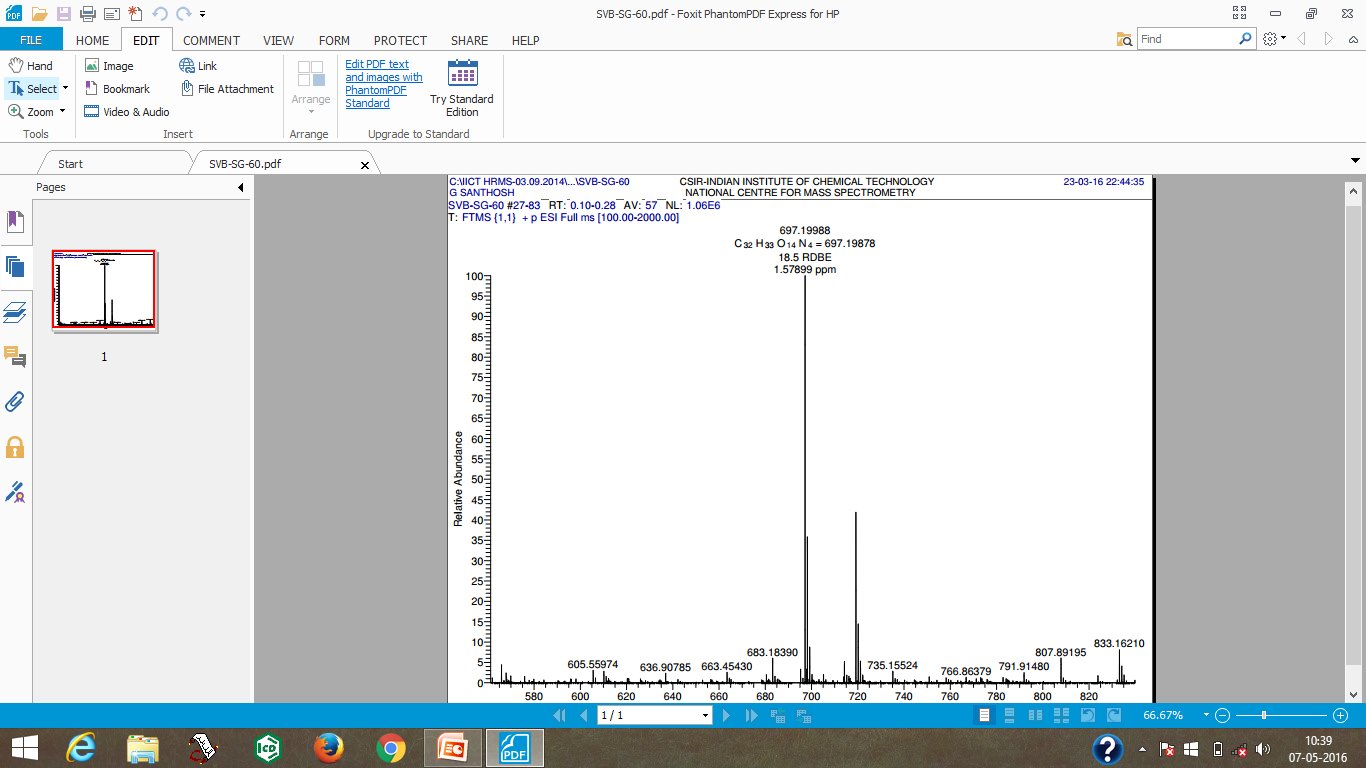
**

**Figure S11.** HRMS of compound **C.**


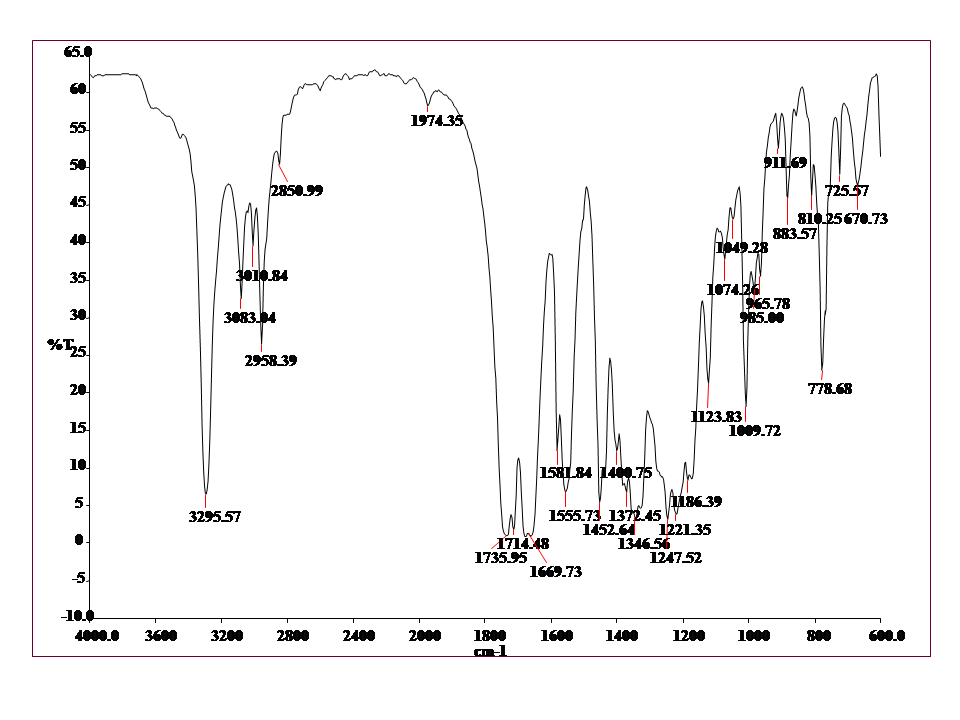


**Figure S12.** FT-IR of compound **C.**

**
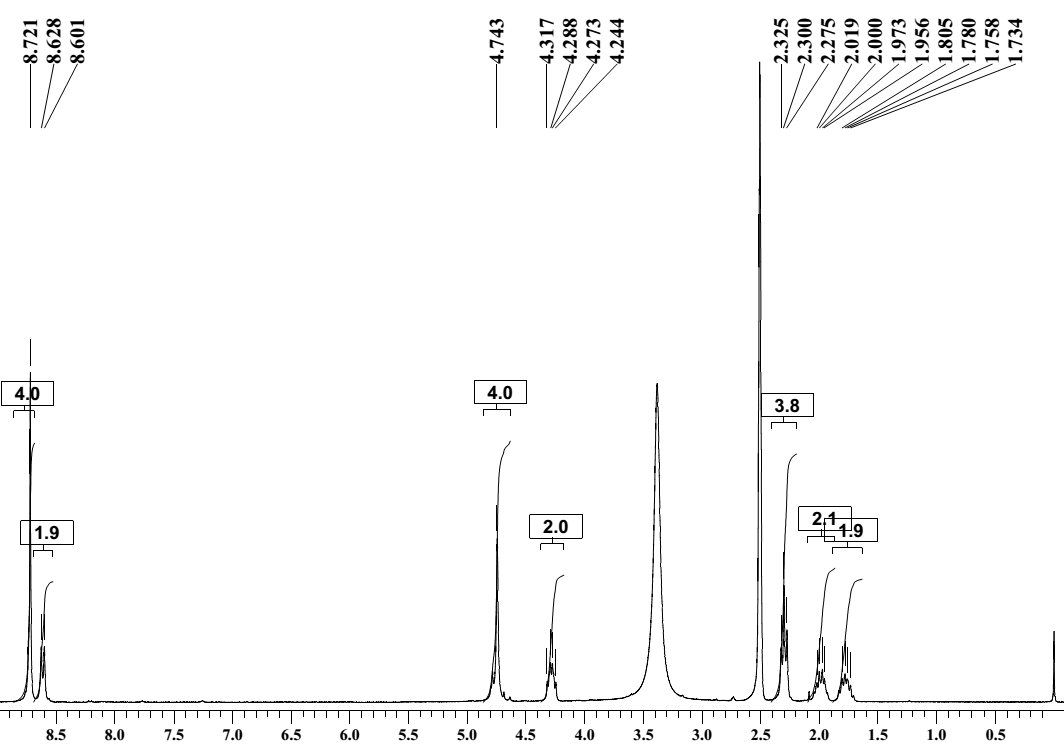
**

**Figure S13.** ^1^H NMRspectra of **NDI-L-Glu** (**1**)**.**

**
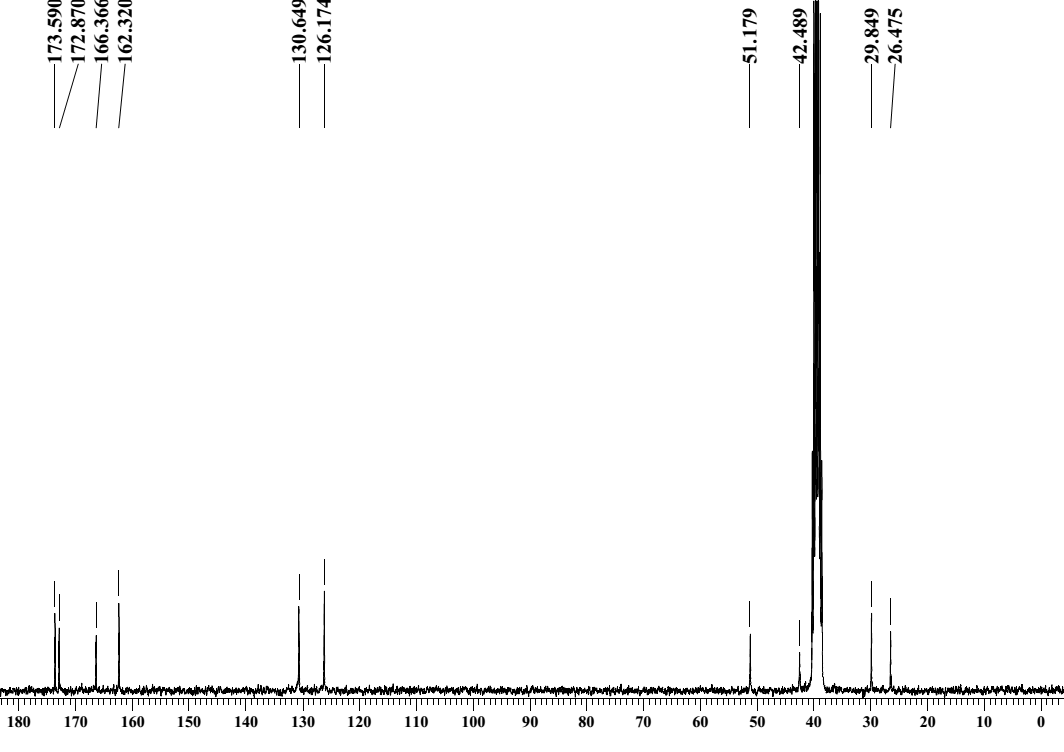
**

**Figure S14.** ^13^C NMR spectra of **NDI-L-Glu** (**1**).

**
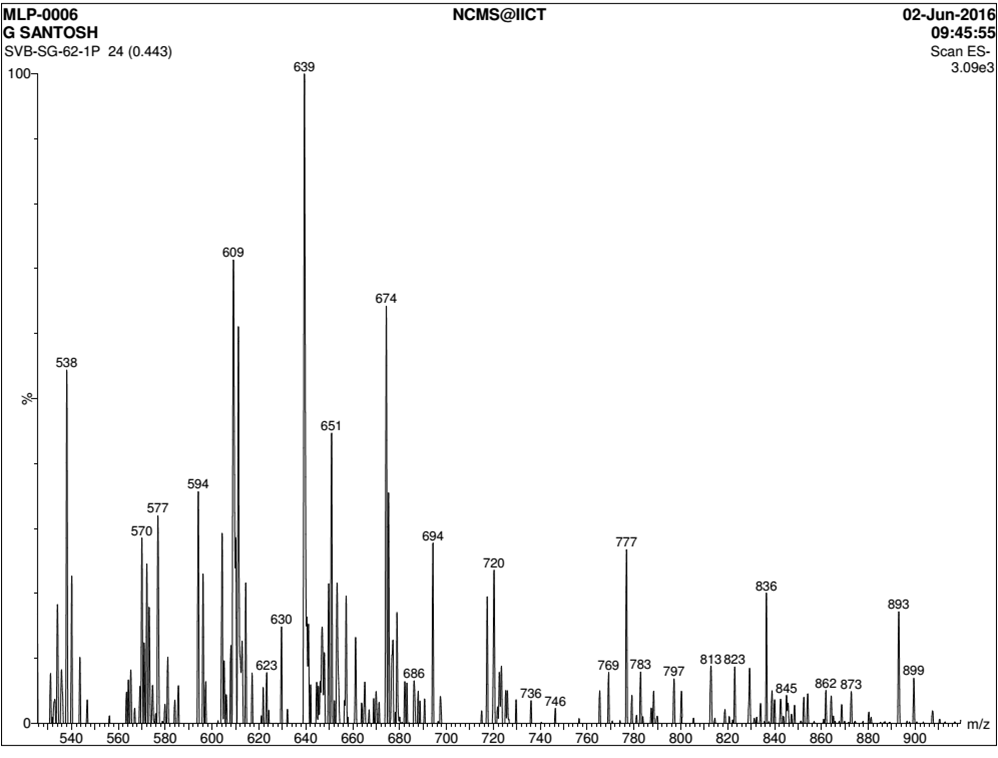
**

**Figure S15.** HRMS of compound **NDI-L-Glu (1)**

**
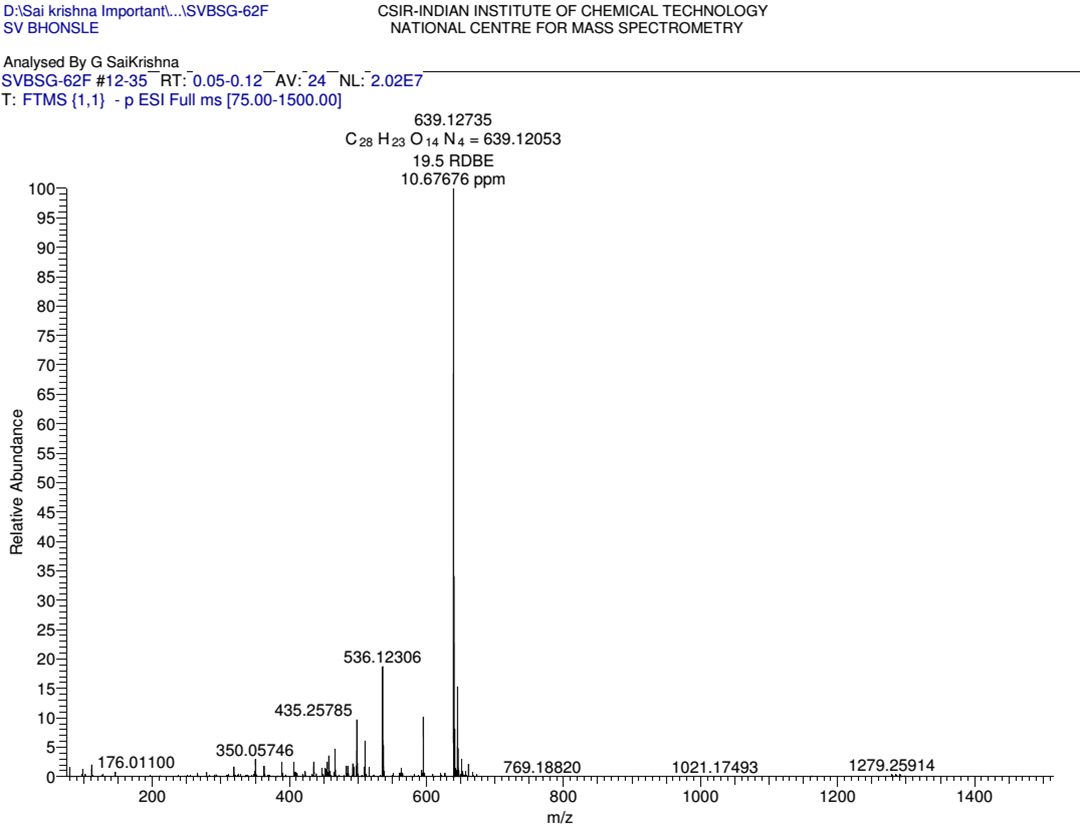
**

**Figure S16.** HRMS of compound **NDI-L-Glu (1)**

**
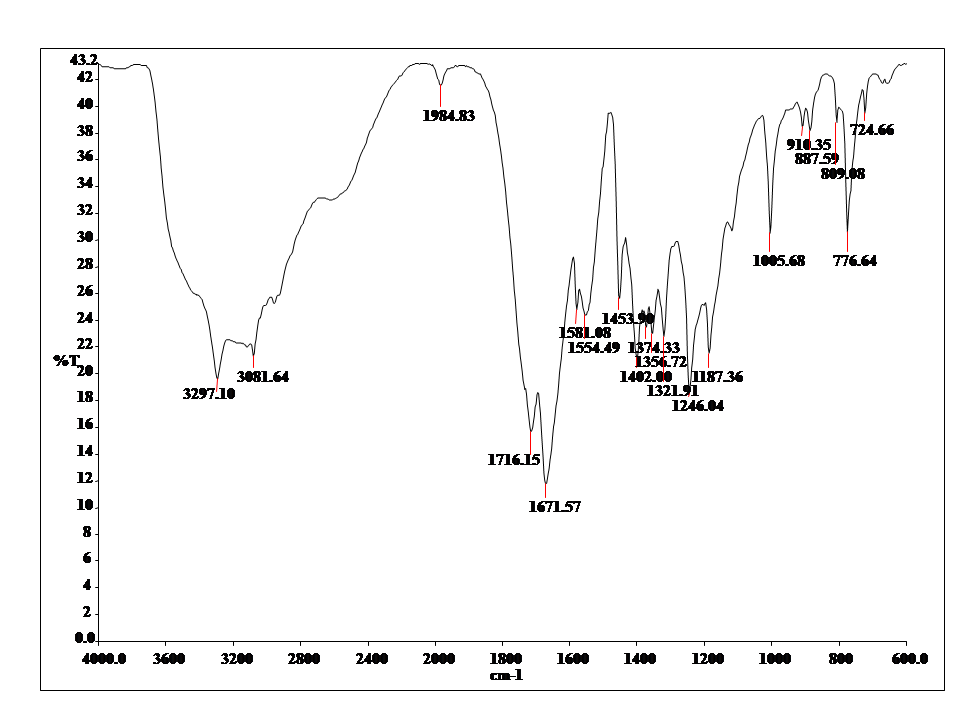
**

**Figure S17.** FT-IR of compound **NDI-L-Glu (1)**

**References**

S1. Kemp JC. Polarized light and its interaction with modulating devices.*Hinds Instruments, Inc, Hillsboro, OR* 1987.
